# Supplementary material for: Bipolar filaments of human nonmuscle myosin 2-A and 2-B have distinct motile and mechanical properties
Source: eLife. 2018 Feb 8;7:e32871. doi: 10.7554/eLife.32871 (PMC5829915; doi:10.7554/eLife.32871)
Supplement: Supplementary file 1. [file elife-32871-supp1.docx]

Table S1. Results of mixing full length NM2-B with NM2-B tail fragments

|  | I_2B,c_ | I_2B,cof_  (∙10^-5^ a.u.) | R_NM2-B_  (∙10^-5^ a.u.) | # of motors per half filament |
| --- | --- | --- | --- | --- |
| 100% NM2-B | 0.66 ± 0.01 | - | 1 ± 0.02 | 30* |
| Mixing ratio 1:1 | - | 0.4 ± 0.01 | 0.61 ± 0.02 | 18 ± 1 |
| Mixing ratio 1:2 | - | 0.31 ± 0.01 | 0.47 ± 0.02 | 14 ± 1 |
| Mixing ratio 1:5 | - | 0.21 ± 0.02 | 0.31 ± 0.02 | 9 ± 2 |

All values are expressed as the mean ± SEM

Mixing ratio – NM2-B:NM2-B tail fragment

*From Billington et al (2013)
