# Supplementary material for: Bipolar filaments of human nonmuscle myosin 2-A and 2-B have distinct motile and mechanical properties
Source: eLife. 2018 Feb 8;7:e32871. doi: 10.7554/eLife.32871 (PMC5829915; doi:10.7554/eLife.32871)
Supplement: Supplementary file 2. [file elife-32871-supp2.docx]

Table S2

|  | Velocity  (nm∙s^-1^) | I_2A,c_  (∙10^-5^ a.u.) | I_2B,c_  (∙10^-5^ a.u.) | I_2A,mix_  (∙10^-5^ a.u.) | I_2B, mix_  (∙10^-5^ a.u.) | Fraction NM2-A |
| --- | --- | --- | --- | --- | --- | --- |
| 100% NM2-B | 43±1 | - | 0.66 ± 0.01 | - | - | 0 |
| Mixing ratio 2:1 | 46±1 | - | - | 0.26±0.01 | 0.64±0.02 | 0.24±0.03 |
| Mixing ratio 1:1 | 43±2 | - | - | 0.21±0.01 | 0.28±0.01 | 0.37±0.06 |
| Mixing ratio 1:2 | 52±1 | - | - | 0.54±0.01 | 0.17±0.01 | 0.72±0.03 |
| 100% NM2-A | 133±7 | 0.85±0.06 | - | - | - | 1±0.09 |

All values are expressed as the mean ± SEM
