# Supplementary material for: Bipolar filaments of human nonmuscle myosin 2-A and 2-B have distinct motile and mechanical properties
Source: eLife. 2018 Feb 8;7:e32871. doi: 10.7554/eLife.32871 (PMC5829915; doi:10.7554/eLife.32871)
Supplement: Supplementary file 3. [file elife-32871-supp3.docx]

Table S3. Effect of phalloidin on the rate of gliding of actin by NM2-A

|  |  |  |  |  |
| --- | --- | --- | --- | --- |
| Single color experiments | Alexa 647 phallodin actin | Atto 538 actin |  |  |
| Velocity (nm∙s^-1^) | 98.8±19.9 | 105±22.6 |  |  |
| Mixed color experiments* | Alexa 647 phallodin actin | Atto 538 actin |  |  |
| Velocity (nm∙s^-1^) | 84.6±28.6 | 86.0±26.7 |  |  |

*In the mixed color experiments, preformed Alexa-Fluor 647-labeled actin filaments were mixed with preformed Atto-538-labeled actin filaments and added to the same flow chamber containing surface-bound NM2-A HMM. The rates in these experiments are somewhat lower than in the text since the assay was conducted at room temperature.
